# Supplementary material for: Epitaxial Growth and Characterization of Nanoscale Magnetic Topological Insulators: Cr-Doped (Bi0.4Sb0.6)2Te3
Source: Nanomaterials (Basel). 2024 Jan 11;14(2):157. doi: 10.3390/nano14020157 (PMC10821443; doi:10.3390/nano14020157)
Supplement: Supplementary file 1 [file nanomaterials-14-00157-s001.zip › nanomaterials-2783552-supplementary.pdf]

nanomaterials

MDPI

Supplementary Documents

Epitaxial Growth and Characterization of Nanoscale Magnetic Topological Insulators: Cr-Doped $(Bi_{0.4}Sb_{0.6})_2Te_3$

Pangihutan Gultom [1], Chia-Chieh Hsu [1], Min Kai Lee [2], Shu Hsuan Su [1,*] and Jung-Chung-Andrew Huang [1,2,3,4,*]

1 Department of Physics, National Cheng Kung University, Tainan 701, Taiwan
2 Instrument Division, Core Facility Center, National Cheng Kung University, Tainan 701, Taiwan
3 Department of Applied Physics, National Kaohsiung University, Kaohsiung 811, Taiwan
4 Taiwan Consortium of Emergent Crystalline Materials, Ministry of Science and Technology, Taipei 10601, Taiwan
* Correspondence: macg0510@yahoo.com.tw (S.H.S.); jcahuang@mail.ncku.edu.tw (J.-C.-A.H.)

Citation: Gultom, P.; Hsu, C.-C.; Lee, M.K.; Su, S.H.; Huang, J.-C.-A. Epitaxial Growth and Characterization of Nanoscale Magnetic Topological Insulators: Cr-Doped $(Bi_{0.4}Sb_{0.6})_2Te_3$. *Nanomaterials* **2024**, *14*, 157. https://doi.org/10.3390/nano14020157

Academic Editor: Yurii K. Gun'ko

Received: 5 December 2023
Revised: 30 December 2023
Accepted: 7 January 2024
Published: 11 January 2024

Copyright: © 2024 by the authors. Licensee MDPI, Basel, Switzerland. This article is an open access article distributed under the terms and conditions of the Creative Commons Attribution (CC BY) license (https://creativecommons.org/licenses/by/4.0/).

Crystal structure and surface morphology study of Cr:BST with Cr/Sb ratio = 0.1-0.3

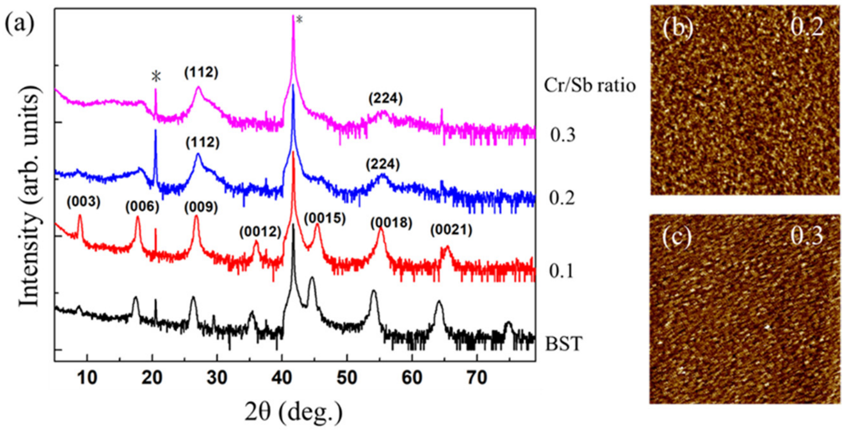

Figure S1. (a) XRD diagrams for growth temperature fixed at 300 °C with varied Cr/Sb ratio (indicated on the right-hand axis). AFM images (3μm x 3μm) for **(b)** Cr/Sb = 0.2 (2μm x 2μm) and **(c)** Cr/Sb = 0.3.

Lattice vibrational study

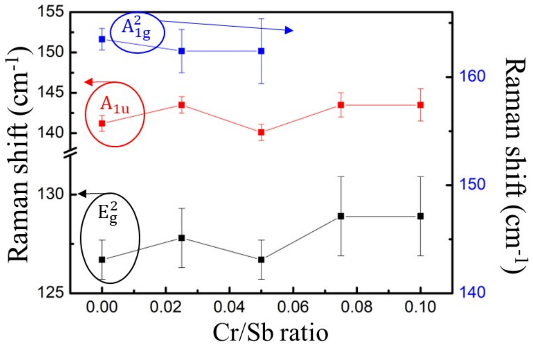

Figure S2. Raman shift vs Cr/Sb ratio for Cr:BST grown at 300 °C with different Cr/Sb ratio of 0, 0.025, 0.05, 0.075, and 0.1.

The shift trends of three Raman signals can be attributed to the Urbach tailing effect, wherein the substitution of some Sb and Bi by Cr atoms leads to the depression of the crystal lattice spacing.[1]

XPS uncertainty

Figure S3 present the Te 3d core levels for both the BST sample and the Cr:BST sample. A distinct shoulder in the Te 3d core level is observed in the Cr:BST sample, which is absent in the BST sample. This feature, highlighted by green arrows in Figure S3, is believed to be associated with the Cr 2p core levels.[2,3]

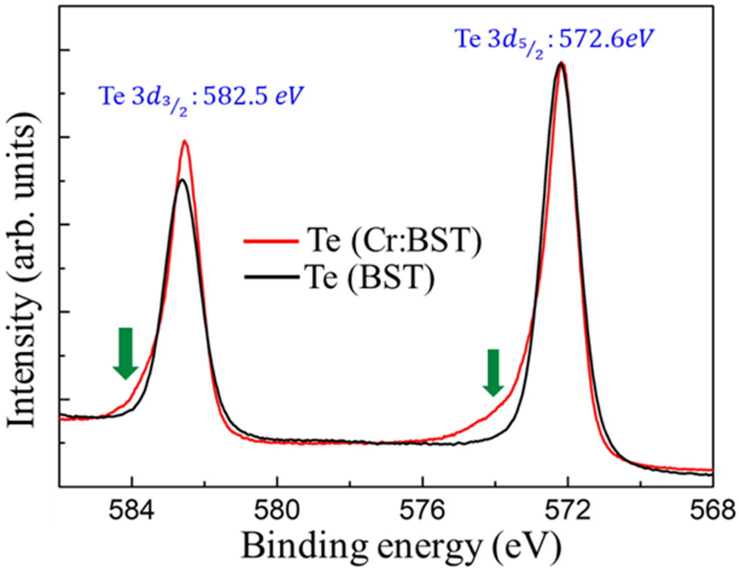

Figure S3. The Te 3d core level for Cr:BST sample with Cr/Sb=0.1 and BST sample.

Magnetic Properties

The established data analysis method for extracting the ferromagnetic signal involves using linear background subtraction to remove the diamagnetic signal from the substrate.[8-10] In Figure S4, the original M-H loop data for the Cr/Sb=0.025 sample at 20K is presented. It is evident that the linear signals at high magnetic fields are dominated by the linear diamagnetic component from the sapphire substrate, but some non-linear effect appears at low magnetic fields.

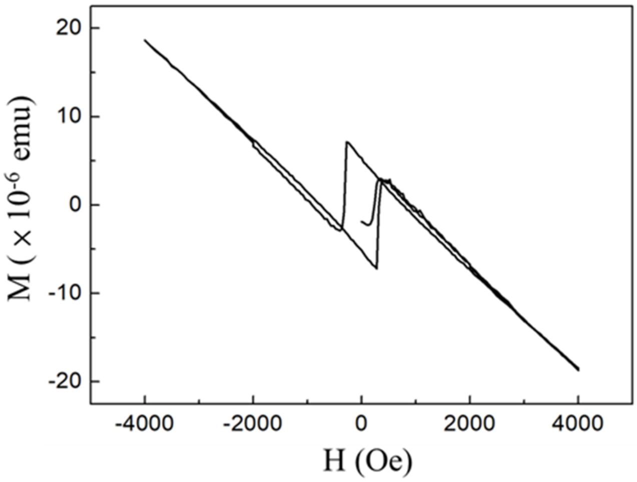

Figure S4. The original SQUID data of Cr/Sb=0.025 sample at 20K.

References

[1] Ho, Y.; Sekwon, N.; Jun, G, G.; Seong, J, J.; Seungmin, H.; and Hoo, J, L. Effects of Sb content (x) on $(Bi_{1-x}Sb_x)_2Te_3$ thermoelectric thin film deposited by Effusion cell evaporator. *J. Nanosci. Nanotechno* **2015**, 15, 10.

[2] Huang, H., Shen, J., Chen, J.Shen, Q.; Lin, G.; Zhu, Z.; Wu, J.; Ma, J.; Yang, H.; Liu, X.; Liu, L.; Guan, D.; Wang, S.; Li, Y.; Liu, C.; Zheng, H.; Lu, Y.; Jia, J. Controllable phase transition of two-dimensional ferromagnetic chromium telluride thin films grown by molecular beam epitaxy. Quantum Front 2023, 2, 12 .

[3] Yu, F.; Yin, Y.; Liu, G.; Tian, Q.; Meng, Q.; Zhao, W.; Wang, K.; Wang, C.; Yang, S.; Wu, D.; Wan, X.; Zhang, Y. Thickness-dependent structural phase transition and self-intercalation of two-dimensional ferromagnetic chromium telluride thin films. Appl. Phys. Lett. 2022,120, 261602.

Disclaimer/Publisher's Note: The statements, opinions and data contained in all publications are solely those of the individual author(s) and contributor(s) and not of MDPI and/or the editor(s). MDPI and/or the editor(s) disclaim responsibility for any injury to people or property resulting from any ideas, methods, instructions or products referred to in the content.
